# Intrauterine insemination as a primary viable option to infertile couples: evaluation of patients in a private center

**DOI:** 10.5935/1518-0557.20190014

**Published:** 2019

**Authors:** Marília Porto Bonow, Ricardo Ditzel Delle Donne, Vinicius Bonato da Rosa, José Augusto Lucca, Cristian Maio Hillesheim, Alessandro Schuffner

**Affiliations:** 1 Gynecology and Obstetrics Post Graduation Program of Federal University of Paraná (UFPR) PR - Brazil; 2 Positivo University (UP). Curitiba - PR - Brazil; 3 Conceber Reproductive Medicine Center. Curitiba - PR - Brazil; 4 Department of Obstetrics and Gynaecology of Federal University of Paraná (UFPR) - Curitiba - PR- Brazil

**Keywords:** Intrauterine insemination, endometrial thickness, recombinant follicle stimulating hormone, sperm motility.

## Abstract

**Objective:**

This study aimed to identify which parameters positively affect the clinical pregnancy rates of IUI cycles and find which couples should opt for IUI.

**Methods:**

This retrospective observational study included 261 patients submitted to 381 IUI cycles with fresh or cryopreserved partner semen (IU-H) from January 2012 to February 2017 in a private center in Curitiba-PR, Brazil.

**Results:**

Idiopathic infertility was the most frequent finding (35.9%). Patients younger than 40 years accounted for 87.9% of the IUI cycles (n=335) and 16.1% of the clinical pregnancies (n=54). The pregnancy rate was three times higher in patients with an endometrium thickness ≥8 mm compared to patients with endometrium thickness <8mm. Sperm motility >55% was linked to higher pregnancy rates (*p*=0.002). Concerning gonadotropins, 159 (48.4%) took rFSH, 127 (38.7%) hMG, and 42 (12.8%) uFSH, with pregnancy rates of 21.3%, 10.4% and 10.5%, respectively.

**Conclusion:**

Patients under 40 years of age with endometrium thickness ≥8 mm, sperm motility >55%, and on rFSH had significantly higher pregnancy rates (*p*<0.05).

## INTRODUCTION

The demand for infertility treatment has grown exponentially in Brazil. Intrauterine insemination (IUI) is a low complexity fertility treatment that is much less invasive and inexpensive when compared to in vitro fertilization (IVF) (Isa *et al*., 2014a). Pregnancy rates after IUI vary widely due to multiple patient-related factors (Isa *et al*., 2014a; 2014b; Allen *et al*., 1985; Asante *et al*., 2013; Nuojua-Huttunen *et al*., 1999; Schuffner *et al*., 2009). IUI can be indicated for patients with different causes of infertility, including cervical factor infertility, ovulatory dysfunction, endometriosis, infertility for immunological causes, and idiopathic infertility (Duran *et al*., 2003).

Some factors may have a decisive role in the outcome of IUI, such as age, ovarian reserve, endometrial thickness, types and doses of gonadotropin, and sperm quality. Although several studies have searched for markers of success in IUI, age remains as the best parameter to assess ovarian function and consequently the response to assisted reproductive technologies (Deatsman *et al*., 2016). Although some authors have linked higher levels of AMH to higher pregnancy rates (Bakas *et al*., 2015), the predictive value of AMH remains questionable in scientific literature (Tremellen & Kolo, 2010).

Endometrial thickness has been associated with higher embryo implantation and IUI success rates. There is no consensus in the literature over an ideal cutoff value, although most studies agree on endometrial thickness greater than 7 mm (Biswas *et al*., 2016; Caetano *et al*., 2005).

IUI without controlled ovarian stimulation is not recommended, as natural cycles offer no clinical advantage. Fertility rates are higher in stimulated cycles (Rashidi *et al*., 2013; Fritz & Speroff, 2011). In several countries, it is mandatory to register IUI procedures. In Denmark, for example, IUI treatments have been registered since 2007 (Malchau *et al*., 2014). REDLARA, the Latin American Network of Assisted Reproduction, reported 6,250 cycles of IUI in 2013 with a birth rate per cycle of 14.9% (Zegers-Hochschild *et al*., 2016). In Brazil, outcomes and indications of IUI are scarce.

The aim of the study was to identify which parameters positively affected the clinical pregnancy rates of IUI cycles in a private assisted reproduction center and evaluate which couples should opt for IUI.

## MATERIAL AND METHODS

### Patients

This retrospective observational study included 261 patients submitted to 381 IUI cycles with fresh or cryopreserved partner semen (IU-H) from January 2012 to February 2017 in a private center in Curitiba-PR, Brazil. Heterologous intrauterine insemination procedures were not included, so as not to affect pregnancy rates.

The local institutional review board approved the study. Patients were not required to give consent due to the retrospective nature of the study.

The included subjects were split into groups based on the cause of infertility, age, endometrial thickness (measured two days before IUI), and anti-Müllerian hormone (AMH) levels. AMH levels were measured in 125 patients, and test results were not considered for IUI. The groups were further divided between fresh *vs.* frozen semen and levels of sperm concentration and motility.

### Controlled ovarian stimulation

Controlled ovarian stimulation was achieved by three different protocols: 1) Injectables, 2) Oral medication + injectables, 3) Oral medication. Group 1 was divided into three subgroups: A) human menopausal gonadotropin (hMG) - Menopur^®^, B) recombinant follicle stimulating hormone (rFSH) - Gonal^®^ or Pergoveris^®^, and C) urinary follicle stimulating hormone (uFSH) Fostimon^®^. Group 2 took clomiphene citrate (CC) followed by the injectable medications cited above. Group 3 took CC or Letrozole^®^. The final outcome considered for all variables was clinical pregnancy.

Serial transvaginal ultrasound examination was performed on the second or third day of the cycle and on the sixth day after the start of controlled ovarian stimulation. The remaining tests varied according to the response each patient had to the stimulation protocol. hCG (Choriomon^®^ or Ovidrel^®^) was administered when the mean diameter of the dominant follicle reached at least 18mm. IUI was scheduled 36h to 40h thereafter. Endometrial thickness was evaluated 48h before IUI, without later analysis. Supplementation of the luteal phase with Utrogestan^®^ or Duphaston^®^ was also performed.

### Semen Processing

Semen samples were processed by density gradient centrifugation or sperm wash according to the 2010 guidelines of the World Health Organization (WHO) for semen processing (WHO, 2010). Sperm wash was performed in samples meeting the following criteria: increased viscosity, concentration <15 million per ml, and motility <32%. Other samples underwent density gradient centrifugation. In both procedures, the volume was measured after collection and concentration and motility were evaluated on Makler chambers with the aid of a light microscope.

After complete liquefaction in density gradient centrifugation, the semen samples were processed with density gradient medium (Isolate, Irvine Scientific, California) and sperm washing medium (Modified HTF Medium HEPES with Gentamicin, Irvine Scientific, California). In the processing technique, 90% and 45% colloidal gradient were used. One mL of the lower phase gradient was transferred into a sterile disposable conical-bottom centrifuge tube using a Pasteur pipette. A second 1 mL layer of the upper phase was gently placed on top of the lower phase. Liquefied semen was gently placed onto the upper phase. The sample was centrifuged for 20 minutes at 516G. The supernatant was discarded, and the pellet was resuspended with a Pasteur pipette in 2.5 mL of HTF. Sperm wash was performed with centrifugation for eight minutes at 516G. Then the supernatant was discarded and the pellet resuspended with a Pasteur pipette in 0.5 mL of HTF. Sperm parameters were then evaluated according to the WHO criteria for IUI.

### Statistical analysis

Data were organized on Microsoft Office Excel 2007^®^ and analyzed on SPSS Statistics 22.0^®^. The results were expressed as frequencies and proportions for categorical and qualitative variables, and as measures of dispersion and central tendency for quantitative variables.

The Mann-Whitney test for quantitative variables was used to assess the relationship between clinical/biological variables and pregnancy success. The non-parametric Shapiro-Wilk test was chosen to test the normality of quantitative variables. The Chi-square or Fisher's exact test was used to test the association between qualitative variables and the outcome of treatment. Statistical significance was attributed to comparisons with a *p*-value <0.05.

Linear logistic regression models were used to determine the relationship between outcomes and the clinical/biological variables. Explanatory variables with a significance level of up to 20% in univariate analysis were included in the logistic regression model. The results were expressed as odds ratios (OR).

Statistical power analysis was performed to verify the ability of tests to reject a false null hypothesis. Software program GPower 3.1 was used in the analysis of statistical power from univariate and multivariate analysis.

## RESULTS

Idiopathic infertility was identified in 35.9% of the cases, followed by ovulatory dysfunction with 26.2% and endometriosis with 15.2%. Other less frequent causes were cervical factor, tubal factor, male factor and multiple factor infertility. There was no statistical difference regarding the cause of infertility and the number of pregnancies (*p*=0.8). Fifty-six (14.6%) of the 381 cycles analyzed resulted in clinical pregnancy.

Patients younger than 40 years accounted for 87.9% (335) of the sample and achieved 54 clinical pregnancies (16.1%). The clinical pregnancy rate was five times higher in younger patients (<40 years) compared to their older peers (>40 years) (*p*=0.043). Among the patients with positive clinical pregnancies, only two were older than 40 years (Table 1). When the patients were split by age (≤29, 30-34, 35-39, and ≥40 years), most pregnancies - 26 (6.8%) - occurred between 35 and 39 years of age (Table 2), but the groups were not statistically different (*p*=0.156).

**Table 1:** Pregnancy rates and patient characteristics

Clinical pregnancy	Positive	Negative	Total	*p*
Age				0.043
<40 years	54	281	335	
≥40 years	2	44	46	
Endometrium thickness				0.016
<8 mm	6	83	89	
≥8 mm	50	242	292	
Sperm				0.71
Frozen	1	10	11	
Fresh	55	315	370	

**Table 2:** Pregnancy rates according to maternal age

Age (years)	Positive	Negative	Total	*p*
≤29	6	23	29	0.156
30 – 34	22	127	149	
35 – 39	26	131	157	
≥40	2	44	46	

Endometrial thickness >8mm was found in 76.6% (292) of the patients. Patients with an endometrial thickness greater than 8 mm were three times more likely to achieve clinical pregnancies than the individuals with an endometrial thickness <8mm (*p*=0.016). Of the 89 patients with endometrial thickness <8mm, only six (6.7%) achieved clinical pregnancies (Table 1).

Fresh semen samples were more frequent (97.1%), and sperm concentration ranged from 2 to 300 million per mL, while sperm motility ranged from 8.1% to 95% after processing. Cryopreserved semen was used in 11 procedures, and only one patient achieved clinical pregnancy. Motility above 55% had higher rates of clinical pregnancy (*p*=0.002).

Of the 381 cycles analyzed, 328 (86.0%) were from Group 1 (injectables) (Table 3), and 42 (11.0%) were from Group 2 (injectables plus oral medication). Nine patients (2.3%) took only oral drugs and two (0.52%) did not use medication. The group given oral medication alone and the individuals undergoing medication-free natural cycles did not achieve clinical pregnancy.

**Table 3:** Stimulation protocols and pregnancy

Protocol	Positive	Negative	*p*
No stimulation	0	2	0.187
Letrozole	0	1	
Letrozole + hMG	0	1	
CC	0	8	
hMG only	12	115	
rFSH only	34	125	
uFSH only	4	38	
CC + hMG	3	20	
CC + rFSH	3	11	
CC + uFSH	0	4	
Total	56	325	

In the group taking only injectables, 159 patients (48.4%) took rFSH, 127 (38.7%) were on hMG, and 42 (12.8%) on uFSH, yielding clinical pregnancy rates of 21.3%, 10.4%, and 10.5%, respectively. Patients on rFSH achieved clinical pregnancy rates 2.5 times higher than the individuals on hMG (*p*=0.02) (Table 4). Six (14.2%) of the 42 patients in Group 2 achieved clinical pregnancies, three with clomiphene citrate and rFSH and three with clomiphene citrate and hMG.

**Table 4:** Pregnancy outcomes for each injectable drug used

Pregnancy	rFSH	hMG	uFSH	*p*
Positive	34	12	4	0.02
Negative	125	115	38	
Total	159	127	42	

## DISCUSSION

It is important to point out that even in patients diagnosed with idiopathic infertility, fertility rates may decline due to lower oocyte quality, fertilization failure or embryo implantation abnormalities (Wolff *et al*., 2013). Advanced maternal age is an established independent negative prognostic factor for live births and clinical pregnancy associated with lower chances of spontaneous or assisted pregnancy (Geisler *et al*., 2017). In the present study, a clear relationship was observed between lower clinical pregnancy rates and advanced maternal age. IUI procedures do not achieve high pregnancy rates in this group. In another similar study carried out in Brazil, advanced maternal age was the only variable significantly correlated with success rates of IUI (Sicchieri *et al*., 2018). Adequate endometrial thickness is widely considered a decisive factor in the outcomes of ART treatments (Wolff et al., 2013). The finding in our study that more than 93% of the patients with endometrium thickness >8 mm did not achieve pregnancy corroborates other studies. In a large study enrolling 2,929 patients submitted to IUI with idiopathic infertility factor, the results plateaued at an endometrial thickness of 10 mm . The literature is still controversial in relation to very thick endometria (>14mm), and some studies suggested that there might be less favorable endometrial characteristics in this group, as well as lower implantation rates (Dietterich *et al*., 2002; Weissman *et al*., 1999). Some studies suggested that the ultrasound aspect of the endometrium (echogenicity) might be linked to higher implantation rates. This was not analyzed in the present study (Alborzi *et al*., 2005).

The link between increased pregnancy rates and increased sperm motility is in agreement with the literature (Duran *et al*., 2003). Few studies have looked into the outcomes of IUI with frozen semen. Our study corroborated the literature, suggesting lower pregnancy rates when frozen semen samples were used in IUI (Dinelli *et al*., 2014). Since our study featured a limited number of patients using frozen semen, more studies are needed to determine whether IUI should be carried out with frozen semen. Since motility is very important to achieving successful outcomes, it has been suggested that the causes of low sperm motility after semen processing, such as varicocele, should be corrected prior to assisted reproduction procedures (Hendin *et al*., 2000).

Although only a few patients did not take injectables in our study, cycles stimulated with injectable or oral medications and injectables only have reportedly greater chances of success. In a study by Malchau *et al*. (2014), 76% of the children born after IUI resulted from controlled ovarian stimulation with CC, FSH or both (Rashidi *et al*., 2013).

A trend toward higher pregnancy rates was observed with rFSH (Demirol & Gurgan, 2007), but the cost-effectiveness of this drug compared to hMG is questionable, since rFSH is more expensive than hMG (Gerli *et al*., 2008). In addition to providing better ovarian response, cycles with rFSH reportedly yield better quality oocytes (Cheon *et al*., 2004). Our study showed better results in stimulated cycles using rFSH, but the medication is not affordable by the standards of most of our patients. Moreover, positive pregnancy outcomes may also be achieved through other stimulation protocols.

## CONCLUSION

IUI is a viable procedure and a good option for the initial treatment of infertile couples, since high pregnancy rates may be achieved when patients are adequately selected.

In this study, IUI patients younger than 40 years with endometrial thickness greater than 8mm had higher clinical pregnancy rates. Endometrial thickness may be an important parameter to be assessed in future research. Sperm motility also had a significant impact on the success of IUI cycles. Further studies are needed to evaluate whether frozen sperm negatively affects pregnancy rates.

Our results suggest that rFSH produces better outcomes than hMG, but more prospective controlled studies are needed to confirm the efficiency of these drugs in IUI.

The decision to perform the most appropriate treatment for each patient should be a priority in ART centers. It is important to carry out studies in each service and compare the findings with data from the literature.

## References

[r1] Alborzi S, Momtahan M, Zolghadri J, Parsanezhad ME (2005). The effect of endometrial pattern and thickness on pregnancy rate
in controlled ovarian hyperstimulation - Intrauterine
Insemination. Med J Islam Repub Iran.

[r2] Allen NC, CM 3rd Herbert, Maxson WS, Rogers BJ, Diamond MP, Wentz AC (1985). Intrauterine insemination: a critical review. Fertil Steril.

[r3] Asante A, Coddington CC, Schenck L, Stewart EA (2013). Thin endometrial stripe does not affect likelihood of achieving
pregnancy in clomiphene citrate/intrauterine insemination
cycles. Fertil Steril.

[r4] Bakas P, Boutas I, Creatsa M, Vlahos N, Gregoriou O, Creatsas G, Hassiakos D (2015). Can anti-Mullerian hormone (AMH) predict the outcome of
intrauterine insemination with controlled ovarian
stimulation?. Gynecol Endocrinol.

[r5] Biswas JK, Bandhu HC, Singh H, Dey M (2016). Relation of endometrial thickness and pregnancy rates in
intrauterine insemination following ovulation induction. Int J Reprod Contracept Obstet Gynecol.

[r6] Caetano JPJ, Cota AMM, Sousa EBA, Lamaita RM, Moraes LAM, Aguiar LPT, Marinho RM (2005). Comparison between three protocols for ovulation Induction in
cycles of intrauterine insemination and related endometrial thickness and
pregnancy rate achieved in each protocol. Rev Bras Ginecol Obstet.

[r7] Cheon KW, Byun HK, Yang KM, Song IO, Choi KH YK (2004). Efficacy of recombinant human follicle-stimulating hormone in
improving oocyte quality in assisted reproductive techniques. J Reprod Med.

[r8] Deatsman S, Vasilopoulos T, Rhoton-Vlasak A (2016). Age and Fertility: A Study on Patient Awareness. JBRA Assist. Reprod.

[r9] Demirol A, Gurgan T (2007). Comparison of different gonadotrophin preparations in
intrauterine insemination cycles for the treatment of unexplained
infertility: a prospective, randomized study. Hum Reprod.

[r10] Dietterich C, Check JH, Choe JK, Nazari A, Lurie D (2002). Increased endometrial thickness on the day of human chorionic
gonadotropin injection does not adversely affect pregnancy or implantation
rates following in vitro fertilization-embryo transfer. Fertil Steril.

[r11] Dinelli L, Courbière B, Achard V, Jouve E, Deveze C, Gnisci A, Grillo JM, Paulmyer-Lacroix O (2014). Prognosis factors of pregnancy after intrauterine insemination
with the husband's sperm: conclusions of an analysis of 2,019
cycles. Fertil Steril.

[r12] Duran HE, Morshedi M, Kruger T, Oehninger S (2003). Intrauterine insemination: a systematic review on determinants of
success. Hum Reprod Update.

[r13] Fritz MA, Speroff L (2011). Clinical Gynecologic Endocrinology and Infertility.

[r14] Geisler ME, Ledwidge M, Bermingham M, McAuliffe M, McMenamin MB, Waterstone JJ (2017). Intrauterine insemination-No more Mr. N.I.C.E.
guy?. Eur J Obstet Gynecol Reprod Biol.

[r15] Gerli S, Bini V, Di Renzo GC (2008). Cost-effectiveness of recombinant follicle-stimulating hormone
(FSH) versus human FSH in intrauterine insemination cycles: a statistical
model-derived analysis. Gynecol Endocrinol.

[r16] Hendin BN, Falcone T, Hallak J, Nelson DR, Vemullapalli S, Goldberg J, Thomas Jr AJ, Agarwal A (2000). The effect of patient and semen characteristics on live birth
rates following intrauterine insemination: a retrospective
study. J Assist Reprod Genet.

[r17] Isa AM, Abu-Rafea B, Alasiri SA, Al-Mutawa J, Binsaleh S, Al-Saif S, Al-Sager A (2014a). Accurate diagnosis as a prognostic factor in intrauterine
insemination treatment of infertile Saudi patients. J Reprod Infertil.

[r18] Isa AM, Abu-Rafea B, Alasiri SA, Binsaleh S, Ismail KH, Vilos GA (2014b). Age, Body mass index, and number of previous trials: are they
prognosticators of intra-uterine-insemination for infertility
treatment?. Int J Fertil Steril.

[r19] Malchau SS, Loft A, Henningsen AKA, Nyboe Andersen A, Pinborg A (2014). Perinatal outcomes in 6,338 singletons born after intrauterine
insemination in Denmark, 2007 to 2012: the influence of ovarian
stimulation. Fertil Steril.

[r20] Nuojua-Huttunen S, Tomas C, Bloigu R, Tuomivaara L, Martikainen H (1999). Intrauterine insemination treatment in subfertility: an analysis
of factors affecting outcome. Hum Reprod.

[r21] Rashidi M, Aaleyasin A, Aghahosseini M, Loloi S, Kokab A, Najmi Z (2013). Advantages of recombinant follicle-stimulating hormone over human
menopausal gonadotropin for ovarian stimulation in intrauterine
insemination: a randomized clinical trial in unexplained
infertility. Eur J Obstet Gynecol.

[r22] Schuffner A, Polleto RS,  Placido T (2009). Intrauterine insemination: a realist alternative in assisted
reproduction. Reprod Clim.

[r23] Sicchieri F, Silva AB, Silva ACJSRE, Navarro PAAS, Ferriani RA, Reis RMD (2018). Prognostic factors in intrauterine insemination
cycles. JBRA Assist Reprod.

[r24] Tremellen K, Kolo M (2010). Serum anti-Mullerian hormone is a useful measure of quantitative
ovarian reserve but does not predict the chances of live-birth
pregnancy. Aust New Zeal J Obstet Gynaecol.

[r25] Weissman A, Gotlieb L, Casper RF (1999). The detrimental effect of increased endometrial thickness on
implantation and pregnancy rates and outcome in an in vitro fertilization
program. Fertil Steril.

[r26] Wolff EF, Vahidi N, Alford C, Richter K, Widra E (2013). Influences on endometrial development during intrauterine
insemination: clinical experience of 2,929 patients with unexplained
infertility. Fertil Steril.

[r27] World Health Organization - WHO (2010). WHO laboratory manual for the examination and processing of human
semen.

[r28] Zegers-Hochschild F, Schwarze JE, Crosby JA, Musri C, Urbina MT, Latin American Network of Assisted Reproduction (REDLARA) (2016). Assisted reproductive techniques in Latin America: The Latin
American Registry, 2013. J Bras Reprod Assist.

